# Development and validation of a nomogram for predicting early rupture and rebleeding risk after intracranial aneurysm embolization

**DOI:** 10.3389/fneur.2026.1727678

**Published:** 2026-03-12

**Authors:** Pu Du, Fen Yu, Guohao Chen, Wenbo Xu

**Affiliations:** 1 Department of Interventional Cerebrovascular Diseases, School of Medicine, The Sixth Affiliated Hospital of South China University of Technology (Nanhai District People’s Hospital of Foshan), Foshan, China; 2 Department of Medical Imaging Center, The Second People’s Hospital of Foshan, Affiliated Foshan Hospital of Guangdong Pharmaceutical University, Foshan, China

**Keywords:** early rebleeding, endovascular embolization, intracranial aneurysm, nomogram model, risk factors, subarachnoid hemorrhage

## Abstract

**Objective:**

To construct and validate a nomogram for predicting early rupture rebleeding risk after intracranial aneurysm embolization, providing a precise clinical assessment tool.

**Methods:**

Clinical data from 274 patients (March 2022–February 2025) were retrospectively analyzed, divided into a training set (*n* = 192) and validation set (*n* = 82) (7:3 ratio). Univariate/multivariate logistic regression identified independent risk factors. The nomogram’s performance was evaluated via ROC curves, calibration curves, and DCA.

**Results:**

Multivariate logistic regression analysis revealed that larger aneurysm diameter, wider neck, higher preoperative Hunt-Hess grade, incomplete embolization, and poor postoperative blood pressure control were independent risk factors for early rupture rebleeding after embolization (all *p* < 0.05). The constructed nomogram demonstrated good calibration and discriminative ability in both the training and validation sets, with C-index values of 0.873 and 0.738, respectively. The areas under the ROC curves (AUC) were 0.870 (95%CI: 0.790–0.951) and 0.739 (95%CI: 0.456–1.000) with corresponding sensitivities and specificities of 0.812, 0.840 and 0.667, 0.902, respectively. Decision curve analysis indicated significant clinical utility within specific threshold probability ranges.

**Conclusion:**

The multifactor nomogram exhibits strong predictive performance, facilitating early identification of high-risk patients and personalized treatment.

## Introduction

Ruptured intracranial aneurysms are the leading cause of spontaneous subarachnoid hemorrhage, associated with high rates of disability and mortality. With the rapid advancement of neuro interventional techniques, endovascular embolization has emerged as a primary treatment modality for intracranial aneurysms due to its minimally invasive nature, safety, and rapid postoperative recovery (1). However, early postoperative rebleeding remains a severe complication, with a relatively low incidence but significantly worsened clinical outcomes upon occurrence. A 2024 meta-analysis including 12,890 patients reported a mortality rate of 35–55% (2). Therefore, accurately identifying risk factors for early rebleeding and establishing an effective predictive model are crucial for reducing mortality and improving clinical prognosis (3).

Current research on risk factors for rebleeding after endovascular embolization of intracranial aneurysms is predominantly limited to single-center retrospective analyses, with inconsistent conclusions across studies. Existing risk prediction models primarily rely on traditional scoring systems or regression equations, which exhibit limited clinical utility and visualization, hindering rapid and accurate risk assessment in clinical practice (4). As an intuitive predictive tool, the nomogram integrates multiple risk factors into a single quantitative index, enabling clinicians to efficiently estimate risk probabilities based on individual patient characteristics. This approach has been widely adopted in oncology, cardiovascular diseases, and other fields (5).

This study aims to systematically collect clinical data from patients undergoing endovascular embolization for intracranial aneurysms, identify independent influence factors for early postoperative rebleeding, and construct a nomogram-based predictive model for predicting the risk of early rupture rebleeding after endovascular embolization in patients with intracranial aneurysms, providing a precise basis for clinical assessment.

## Materials and methods

### Study subjects

A total of 274 patients who underwent endovascular embolization for intracranial aneurysms in the Department of Neurosurgery between March 2022 and February 2025 were retrospectively enrolled. The inclusion criteria were as follows: (1) diagnosis of intracranial aneurysm confirmed by digital subtraction angiography (DSA), computed tomography angiography (CTA), or magnetic resonance angiography (MRA). (2) undergoing endovascular embolization therapy; and availability of complete clinical data. The exclusion criteria included: (1) concomitant severe cerebrovascular diseases (e.g., arteriovenous malformation, moyamoya disease); severe dysfunction of major organs (heart: NYHA class IV; liver: Child-Pugh class C; kidney: eGFR <30 mL/min/1.73m^2^). (2) incomplete medical records that could affect data analysis. Using a random number table, the patients were allocated into a training set (*n* = 192) and a validation set (*n* = 82) at a ratio of 7:3. All surgeries adopted the femoral artery approach as the standard interventional access, with no radial artery approach used.

### Surgical procedure and team

All endovascular embolization procedures were performed by a fixed team of three senior neurointerventionalists, each with more than 10 years of experience in neurointerventional surgery. A standardized institutional protocol was followed for all cases to minimize technical variability.

### Data collection

Comprehensive clinical data were systematically collected. Demographic characteristics included: age and sex. Comorbidity history: history of hypertension, diabetes mellitus, tobacco use and alcohol consumption. Aneurysm characteristics: location, maximum diameter, morphology (regular: saccular with smooth surface and clear neck-body distinction; irregular: fusiform, lobulated saccular, or with mural thrombus, confirmed by CTA/MRA/DSA) and neck width. Clinical assessment indices: preoperative Hunt-Hess grade and modified Fisher grade. Surgical and postoperative parameters: embolization materials (e.g., coils, stents), embolization extent (Raymond-Roy classification), postoperative antiplatelet/anticoagulant therapy (improper antiplatelet use defined as non-adherence to the standardized regimen [clopidogrel 75 mg/d for 3 months + aspirin 100 mg/d for 6 months] or premature discontinuation < 1 month), blood pressure management (poor control defined as systolic blood pressure >140 mmHg sustained for >24 h based on continuous arterial monitoring), occurrence of cerebral vasospasm (diagnosed based on clinical symptoms such as new-onset headache or neurological deficit, combined with imaging evidence of >50% cerebral artery narrowing on DSA or CTA/MRI), and development of hydrocephalus.

### Definition of early postoperative rupture rebleeding

Patients were closely monitored for clinical symptoms and signs postoperatively. Early rupture rebleeding was defined as rebleeding occurring within 7 days after endovascular embolization, determined based on neuroimaging examinations including computed tomography (CT) and DSA. A diagnosis of early postoperative rupture rebleeding was established if the following criteria were met: sudden onset of severe headache, vomiting, or aggravated disturbance of consciousness. CT evidence of expanded subarachnoid hemorrhage or new hemorrhagic foci; and exclusion of other potential causes. Taking early rupture and rebleeding after surgery as the dependent variable (no = 0, yes = 1) (Table 1).

**Table 1:** Variable assignment methods.

Variable	Meaning	Assignment
X1	Aneurysm diameter	Continuous variable
X2	Neck width of aneurysm	Continuous variable
X3	Pre-operative Hunt-Hess grade	Grade I-II = 0, Grade III-V = 1
X4	Embolization degree	Total occlusion = 1, partial occlusion = 0
X5	Post-operative systolic blood pressure	Continuous variable
Y	Early Post-operative rupture and rebleeding	Yes = 1, No = 0

### Statistical analysis

Statistical analyses were performed using SPSS 26.0 and R 4.1.3 software. R packages used included: “caret” (version 6.0–94) for dataset splitting, “rms” (version 6.7–1) for nomogram construction, “pROC” (version 1.18.4) for ROC curve analysis, and “DCA” (version 3.0) for decision curve analysis. Continuous variables conforming to a normal distribution were expressed as mean ± standard deviation (mean ± SD), and intergroup comparisons were conducted using the independent samples *t* -test. Categorical variables were presented as frequencies and percentages, and intergroup comparisons were performed using the chi-square (*χ*^2^) test or Fisher’s exact probability test, as appropriate.

Univariate analysis was employed to screen for factors with *p* < 0.05, which were subsequently incorporated into multivariate logistic regression analysis. To address potential overfitting (attributable to 22 rebleeding events and 5 predictors), LASSO regression (R “glmnet” package, version 4.1–6, *λ* = 0.023) was performed to shrink coefficients, and all 5 predictors remained significant. Bootstrap validation with optimism correction was used to obtain realistic performance estimates. A nomogram model was constructed using the “rms” package in R software. The predictive performance of the model was evaluated by the receiver operating characteristic (ROC) curve, generated using the “pROC” package, and the concordance index (C-index) was calculated. Internal validation of the model was performed using two methods: Bootstrap validation (1,000 resamples) and 5-fold cross-validation. Calibration curves were plotted to assess model calibration, and the mean C-index from cross-validation was calculated to confirm robustness. Decision curve analysis (DCA) was conducted using the “DCA” package to evaluate the clinical utility of the model. A two-sided *p* < 0.05 was considered statistically significant.

## Results

### Baseline characteristics of patients in the training group and the validation group

No statistically significant differences were found in age, gender, history of underlying diseases, aneurysm-related characteristics, surgery-and postoperative-related information between the patients in the training set and the validation set (all *p* > 0.05), indicating comparability between the two groups (Table 2). In the training set (*n* = 192), early rebleeding occurred in 22 patients (11.5%). In the validation set (*n* = 82), early rebleeding occurred in 9 patients (11.0%). The distribution of rebleeding events was comparable between the two sets (*χ*^2^ = 0.023, *p* = 0.879).

**Table 2:** Comparison of baseline characteristics between the training set (modeling set) and validation set.

Clinical characteristics	Training set (*n* = 192)	Validation set (*n* = 82)	*t* or *χ*^2^ value	*p*
Age (years)	51.32 ± 10.23	52.15 ± 10.58	0.608	0.542
Sex (Male/Female)	107 (55.73%)/85 (44.27%)	43 (52.44%)/39 (47.56%)	0.403	0.524
History of hypertension (Yes/No)	78 (40.63%)/114 (59.38%)	33 (40.24%)/49 (59.76%)	0.004	0.952
History of diabetes (Yes/No)	36 (18.75%)/156 (81.25%)	15 (18.29%)/67 (81.71%)	0.007	0.929
History of smoking (Yes/No)	65 (33.85%)/127 (66.15%)	28 (34.15%)/54 (65.85%)	0.002	0.962
History of alcohol consumption (Yes/No)	58 (30.21%)/134 (69.79%)	25 (30.49%)/57 (69.51%)	0.002	0.964
Aneurysm location (Anterior circulation/Posterior circulation)	139 (72.40%)/53 (27.60%)	61 (74.39%)/21 (25.61%)	0.115	0.733
Aneurysm diameter (mm)	6.52 ± 2.31	6.85 ± 2.56	1.047	0.295
Aneurysm morphology (Regular/Irregular)	110 (57.29%)/82 (42.71%)	49 (59.76%)/33 (40.24%)	0.143	0.705
Neck width of aneurysm (mm)	3.56 ± 1.25	3.89 ± 1.34	1.958	0.051
Pre-operative Hunt-Hess grade (Grade I-II/Grade III-V)	139 (72.40%)/53 (27.60%)	59 (71.95%)/23 (28.05%)	0.005	0.939
Pre-operative modified Fisher grade (Grade 1-2/Grade 3–4)	129 (67.19%)/63 (32.81%)	53 (64.63%)/29 (35.37%)	0.168	0.681
Embolization materials (Coils/Scaffold-assisted coils/Liquid embolic agents)	99 (51.56%)/70 (36.46%)/23 (11.98%)	42 (51.22%)/28 (34.15%)/12 (14.63%)	0.404	0.817
Degree of embolization (complete embolization/partial embolization)	131 (68.23%)/61 (31.77%)	53 (64.63%)/29 (35.37%)	0.336	0.561
Post-operative use of anti-platelet drugs (Yes/No)	110 (57.29%)/82 (42.71%)	45 (54.88%)/37 (45.12%)	0.136	0.712
Post-operative use of anticoagulant drugs (Yes/No)	41 (21.35%)/151 (78.65%)	15 (18.29%)/67 (81.71%)	0.331	0.565
Post-operative systolic blood pressure (mmHg)	125.56 ± 10.23	126.12 ± 10.56	0.411	0.681
Post-operative diastolic blood pressure (mmHg)	75.32 ± 8.15	76.05 ± 8.32	0.571	0.568
Post-operative complication of cerebral vasospasm (Yes/No)	30 (15.63%)/162 (84.38%)	13 (15.85%)/69 (84.15%)	0.002	0.961
Post-operative complication of hydrocephalus (Yes/No)	24 (12.50%)/168 (87.50%)	12 (14.63%)/70 (85.37%)	0.229	0.632

### Univariate analysis of early rupture and rebleeding after embolization in patients with intracranial aneurysms

Univariate analysis was conducted in the training set, and the results showed that a history of hypertension, a history of smoking, a history of alcohol consumption, aneurysms located in the posterior circulation, larger aneurysm diameter, irregular aneurysm morphology, wider aneurysm neck, higher preoperative Hunt-Hess grade, higher preoperative modified Fisher grade, incomplete embolization, improper use of anti-platelet drugs after surgery, poor blood pressure control after surgery, concurrent cerebral vasospasm, and concurrent hydrocephalus were associated with early rupture and rebleeding after embolization in patients with intracranial aneurysms (all *p* < 0.05) in Table 3.

**Table 3:** Univariate analysis of early rupture and rebleeding in the training set (modeling set).

Clinical characteristics	Rebleeding group (*n* = 22)	Non-rebleeding group (*n* = 170)	*t* or *χ*^2^ value	*p*
Age (years)	58.25 ± 9.56	50.56 ± 10.12	3.373	0.001
Sex (Male/Female)	12 (54.55%)/10 (45.45%)	95 (55.88%)/75 (44.12%)	0.014	0.905
History of hypertension (Yes/No)	16 (72.73%)/6 (27.27%)	62 (36.47%)/108 (63.53%)	10.615	0.001
History of diabetes (Yes/No)	5 (22.73%)/17 (77.27%)	31 (18.24%)/139 (81.76%)	0.047	0.827
History of smoking (Yes/No)	14 (63.64%)/8 (36.36%)	51 (30.00%)/119 (70.00%)	9.841	0.002
History of alcohol consumption (Yes/No)	12 (54.55%)/10 (45.45%)	46 (27.06%)/124 (72.94%)	6.980	0.008
Aneurysm location (Anterior circulation/Posterior circulation)	8 (36.36%)/14 (63.64%)	131 (77.06%)/39 (22.94%)	16.142	<0.001
Aneurysm diameter (mm)	9.56 ± 3.21	8.12 ± 2.15	2.773	0.006
Aneurysm morphology (Regular/Irregular)	6 (27.27%)/16 (72.73%)	104 (61.18%)/66 (38.82%)	9.151	0.003
Neck width of aneurysm (mm)	5.12 ± 1.56	4.25 ± 1.02	3.513	<0.001
Pre-operative Hunt-Hess grade (Grade I-II/Grade III-V)	12 (54.55%)/10 (45.45%)	127 (74.71%)/43 (25.29%)	3.961	0.046
Pre-operative modified Fisher grade (Grade 1-2/Grade 3–4)	10 (45.45%) / 12 (54.55%)	119 (70.00%) / 51 (30.00%)	10.234	<0.001
Embolization materials (Coils/Scaffold-assisted coils/Liquid embolic agents)	11 (50.00%)/7 (31.82%)/4 (18.18%)	88 (51.76%)/63 (37.06%)/19 (11.18%)	0.956	0.619
Degree of embolization (Complete embolization/Partial embolization)	8 (36.36%)/14 (63.64%)	123 (72.35%)/47 (27.65%)	8.748	0.003
Post-operative use of anti-platelet drugs (Yes/No)	16 (72.73%)/6 (27.27%)	94 (55.29%)/76 (44.71%)	3.638	0.003
Post-operative use of anticoagulant drugs (Yes/No)	6 (27.27%)/16 (72.73%)	35 (20.59%)/135 (79.41%)	0.196	0.657
Post-operative systolic blood pressure (mmHg)	135.56 ± 12.23	123.56 ± 9.56	5.354	<0.001
Post-operative diastolic blood pressure (mmHg)	78.32 ± 8.15	74.56 ± 7.56	2.157	0.031
Post-operative complication of cerebral vasospasm (Yes/No)	12 (54.55%)/10 (45.45%)	18 (10.59%)/152 (89.41%)	25.312	<0.001
Post-operative complication of hydrocephalus (Yes/No)	11 (50.00%)/11 (50.00%)	13 (7.65%)/157 (92.35%)	28.191	<0.001

### Multivariate logistic regression analysis of early rupture and rebleeding after embolization in patients with intracranial aneurysms

The factors with *p* < 0.05 in the univariate analysis were used as covariates for multivariate logistic regression analysis. The results showed that larger aneurysm diameter, wider aneurysm neck, higher preoperative Hunt-Hess grade, incomplete embolization, and poor postoperative blood pressure control were independent risk factors for early rupture and rebleeding after embolization in patients with intracranial aneurysms (all *p* < 0.05) in Table 4.

**Table 4:** Multivariate logistic regression analysis of early rupture and rebleeding after embolization in patients with intracranial aneurysms.

Factors	*β*	SE	Wald	*p*	OR	95%CI
Aneurysm diameter	0.236	0.114	4.255	0.039	1.266	1.012–1.584
Neck width of aneurysm	0.782	0.253	9.560	0.002	2.185	1.331–3.587
Pre-operative Hunt-Hess grade	2.238	0.995	5.057	0.025	9.371	1.333–65.888
Embolization degree	−3.358	1.001	11.250	0.001	0.035	0.005–0.248
Post-operative systolic blood pressure	0.067	0.027	6.298	0.012	1.070	1.015–1.128

### Establishment of a nomogram prediction model for early rupture and rebleeding after embolization in patients with intracranial aneurysms

**Figure 1:**
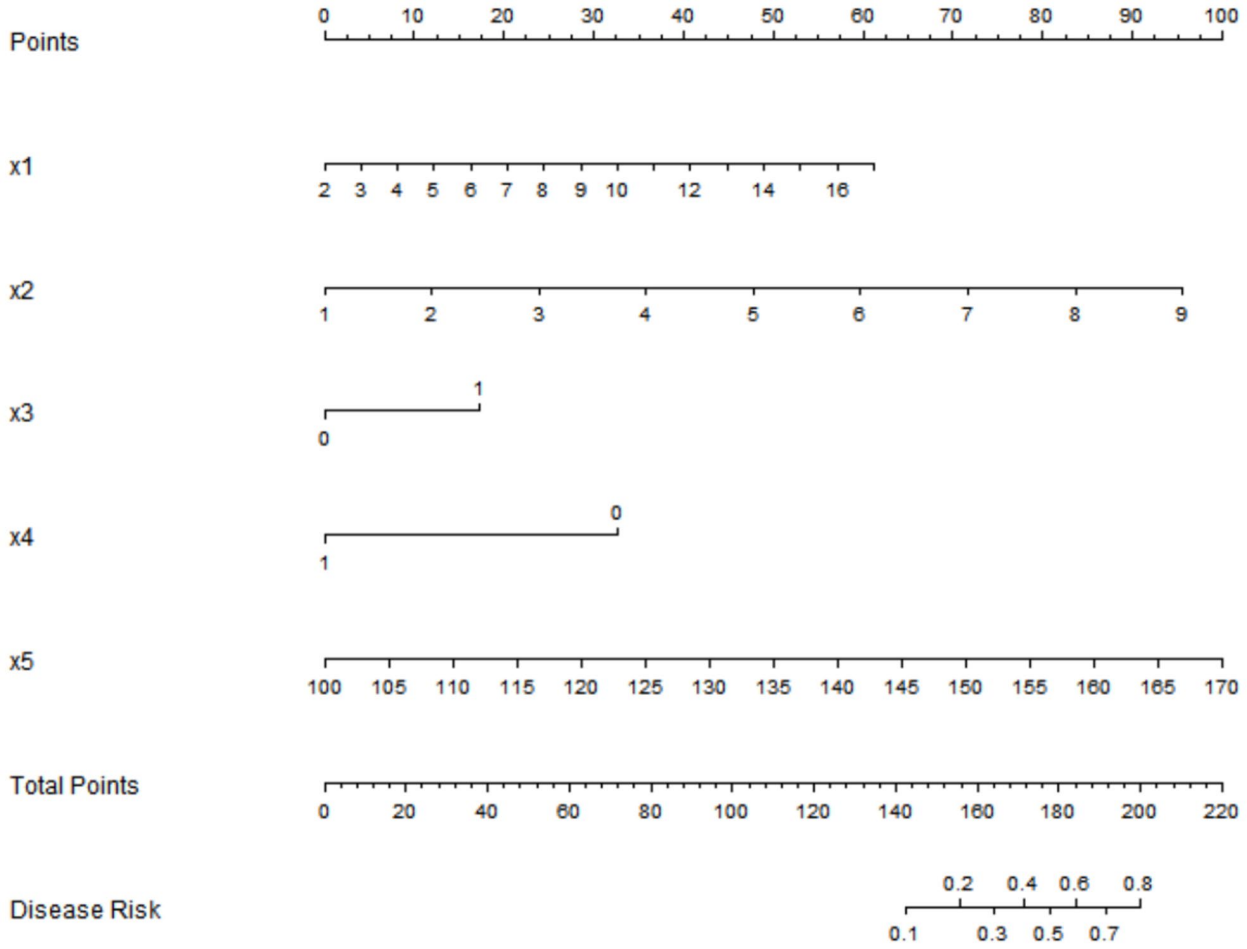
Nomogram prediction model for early rupture and rebleeding after intracranial aneurysm embolization. X1: Aneurysm diameter (mm); X2: Neck width of aneurysm (mm); X3: Pre-operative Hunt-Hess grade (0 = Grade I–II, 1 = Grade III–V); X4: Embolization degree (1 = Complete embolization, 0 = Partial embolization); X5: Post-operative systolic blood pressure (mmHg). Total points: Sum of scores from X1–X5; Disease risk: Predicted probability of early rebleeding.

Based on the independent risk factors identified by multivariate logistic regression analysis, a nomogram prediction model for early rupture and rebleeding after embolization in patients with intracranial aneurysms was constructed. Scores were assigned to each independent risk factor in the model, and the total score for predicting early rupture and rebleeding after surgery was calculated, which was reflected by the probability of predicting early rupture and rebleeding after surgery. The higher the total score, the higher the accuracy of predicting early rupture and rebleeding after surgery in patients in Figure 1. To facilitate clinical application, we provide a hypothetical case example: A 55-year-old female with a 6.8 mm-diameter aneurysm, 4.2 mm neck width, Pre-operative Hunt-Hess grade III (X3 = 1), partial embolization (X4 = 0), and post-operative systolic blood pressure of 138 mmHg. Corresponding points: X1 = 32, X2 = 45, X3 = 60, X4 = 0, X5 = 28. Total score = 165, predicting an early rebleeding risk of ~35%–a high-risk patient requiring strict blood pressure control and close monitoring.

### Evaluation and validation of a nomogram prediction model for early rupture and rebleeding after embolization in patients with intracranial aneurysms

**Figure 2:**
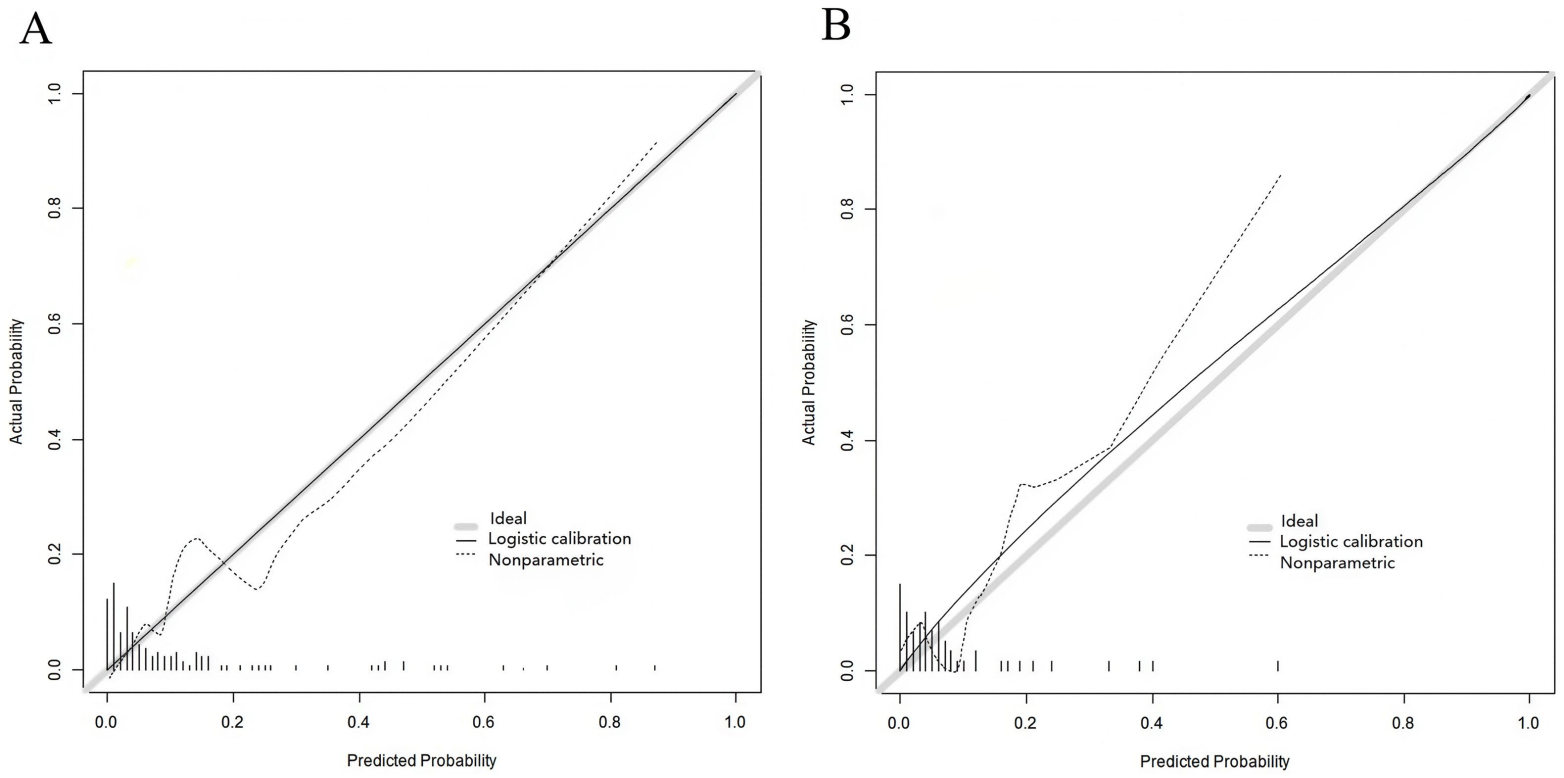
Calibration curves of the nomogram model: **(A)** Training set; **(B)** Validation set. The *x*-axis represents predicted probability of early rebleeding, and the *y*-axis represents actual probability. The solid line indicates ideal calibration (predicted = actual), and the dashed line indicates model calibration.

**Figure 3:**
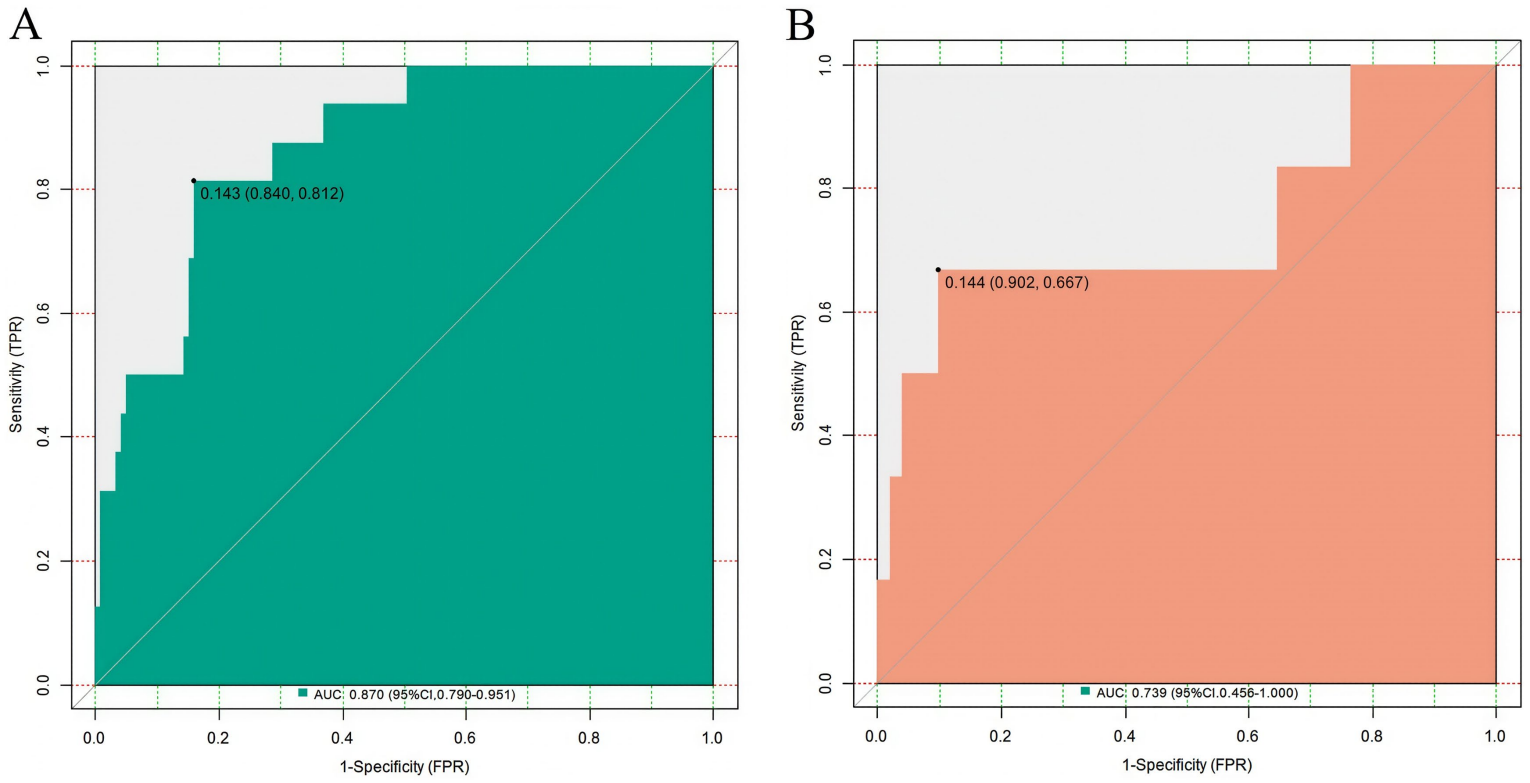
Receiver operating characteristic curves of the nomogram for predicting early rebleeding. **(A)** Training set; **(B)** Validation set. The *x*-axis represents 1 - specificity (false positive rate), and the *y*-axis represents sensitivity (true positive rate). The diagonal dashed line indicates the reference line of no discrimination (AUC = 0.5). The area under the curve for each set is displayed within the graph.

In the training set and the validation set, the C-index values of the nomogram model were 0.873 and 0.738, respectively. The calibration curves showed a good agreement between the predicted values and the actual values. The results of the Hosmer-Lemeshow test were *χ*^2^ = 10.269, *p* = 0.246 and *χ*^2^ = 6.703, *p* = 0.568, respectively. The ROC curves indicated that in the training set and the validation set, the AUCs of the nomogram model for predicting early rupture and rebleeding after embolization in patients with intracranial aneurysms were 0.870 (95%CI: 0.790–0.951) and 0.739 (95%CI: 0.456–1.000) respectively, and the sensitivities and specificities were 0.812, 0.840 and 0.667, 0.902, respectively. The calibration curve is shown in Figure 2, and the ROC curve is shown in Figure 3.

### Decision curve analysis of the nomogram prediction model for early rupture and rebleeding after embolization in patients with intracranial aneurysms

**Figure 4:**
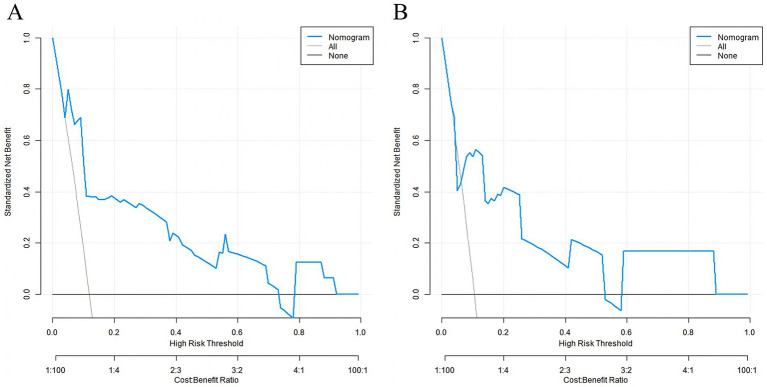
Decision curve analysis of the nomogram for clinical utility assessment. **(A)** Training set; **(B)** Validation set. The *x*-axis represents the threshold probability for clinical intervention. The *y*-axis represents the net benefit. The solid black line (‘Nomogram’) plots the net benefit of using the nomogram for decision-making across threshold probabilities. The grey line (‘All’) assumes all patients experience rebleeding, and the horizontal dashed line (‘None’) assumes no patients experience rebleeding.

Decision curve analysis demonstrated that when the threshold probability was between 0.06 and 0.75, the nomogram had significant clinical net benefit. Key net benefit values at critical thresholds were: 0.10 (10% risk) = 0.062, 0.15 (15% risk) = 0.098, 0.20 (20% risk) = 0.113. Compared with the PHASES score (a widely used tool for aneurysm rupture risk prediction, net benefit at 15% risk = 0.045), our nomogram showed higher clinical utility for post-embolization rebleeding prediction (Figure 4).

## Discussion

Intracranial aneurysms are cerebrovascular diseases that pose a serious threat to human life and health, with extremely high mortality and disability rates after rupture and hemorrhage (6). As one of the main current treatment methods, endovascular embolization can effectively occlude the aneurysm cavity. However, early postoperative rupture and rehemorrhage remains a critical and dangerous complication affecting the prognosis of patients, with an incidence of approximately 2.2–7.4% (7). The occurrence of such complications is not only related to surgical operations but also closely associated with the patients’ own anatomical characteristics, underlying diseases, and peri-operative management (8). However, currently, there is a lack of precise risk assessment tools in clinical practice. In most cases, the judgment relies on the doctors’ experience, resulting in the co-existence of insufficient identification of high-risk patients and over-intervention (9). Therefore, constructing a prediction model based on multi-dimensional clinical features to achieve a quantitative assessment of the early rehemorrhage risk after embolization is of great significance for optimizing clinical decision-making and improving the patients’ prognosis (10). In this study, by incorporating key factors such as aneurysm morphological indicators (diameter, neck width), clinical grading (Hunt-Hess grade), surgical effectiveness (degree of embolization), and peri-operative management (blood pressure control), the aim was to establish the first nomogram model integrating multi-dimensional clinical and surgical factors for early rehemorrhage after embolization. This model can not only integrate multi-variable information for individualized risk prediction but also improve the assessment efficiency of clinicians through visual tools, providing a basis for refined postoperative management.

This study confirmed that a larger aneurysm diameter and a wider aneurysm neck width were independent risk factors for early postoperative rebleeding, which was consistent with the results of previous studies. Larger aneurysms have higher wall tension (consistent with Laplace’s law) and are often accompanied by intra-aneurysmal turbulence and thrombus formation (11). After embolization, the supporting pressure of coils on the aneurysm wall is unevenly distributed, which easily leads to the rupture of the weak part of the aneurysm wall due to continuous blood flow impact [(11)]. The aneurysm neck width directly affected the packing density of the embolization material. Patients with a wide aneurysm neck had difficulty achieving dense embolization, and the residual aneurysm cavity might become a “target” for blood flow impact, increasing the risk of rebleeding (12).

### Preoperative severity of illness

The core value of Hunt-Hess grading. The risk of postoperative rebleeding was significantly increased in patients with a preoperative Hunt-Hess grade ≥ III, suggesting that severe early neurological deficits and intracranial environmental disorders after bleeding (such as cerebral vasospasm and increased intracranial pressure caused by subarachnoid hemorrhage) might weaken the repair ability of the vascular wall (13). These patients often had more complex hemodynamic changes, delayed repair of vascular endothelial cells after embolization, and were prone to early rebleeding (14).

Surgical quality and perioperative management: the crucial roles of embolization degree and blood pressure control. Incomplete embolization is an important inducement for postoperative rebleeding. The residual aneurysm cavity continuously endures blood flow impact, and especially during blood pressure fluctuations, it may lead to coil compression or displacement (15). In this study, it was found that the risk of rebleeding in patients with poor postoperative blood pressure control was 3.2 times that of those with good blood pressure control. Notably, diastolic blood pressure was significantly associated with early rebleeding in univariate analysis but excluded from the multivariate model due to high collinearity with systolic blood pressure [variance inflation factor (VIF) = 3.87], which avoided potential model instability. We evaluated multicollinearity among the five independent predictors using variance inflation factor (VIF): aneurysm diameter (1.82), neck width (2.15), Pre-operative Hunt-Hess grade (1.36), embolization degree (1.28), and post-operative systolic blood pressure (1.57). All VIF values <3 confirm no significant multicollinearity, ensuring model stability. The hypertensive state can directly increase the shear stress on the aneurysm wall [as demonstrated by computational fluid dynamics studies (16)], offsetting the mechanical protective effect of the embolization material and increasing rebleeding risk (16, 17). This suggests that strict postoperative blood pressure management should be the core intervention measure for high-risk patients.

The nomogram demonstrated robust discriminative ability, with a C-index of 0.873 in the training set and 0.738 in the validation set, and corresponding AUCs of 0.870 (95% CI: 0.790–0.951) and 0.739 (95% CI: 0.456–1.000). The notably wide confidence interval in the validation set reflects statistical uncertainty due to the limited number of rebleeding events, underscoring the need for validation in larger cohorts. Although sensitivity decreased in the validation set (0.667 vs. 0.812 in training), the high specificity (0.902) supports its reliability in identifying high-risk patients. Decision curve analysis further confirmed that within a specific range of threshold probabilities, the clinical net benefit of this model was significantly higher than that of traditional empirical judgment, suggesting high clinical application value. By integrating multiple risk factors into a visual chart, the model enables clinicians to quickly calculate the risk score according to the specific indicators of patients and intuitively evaluate the risk of rebleeding, providing a quantitative basis for the formulation of personalized treatment plans (18).

The reasons for not conducting external validation and the limitations of the study are as follows. Although the internal validation efficacy of the model is ideal, external validation has not been carried out, which is mainly restricted by the following factors: Heterogeneity of multicenter data: Significant differences exist among different centers in embolization techniques (such as coil packing density and stent usage ratio) and postoperative management (anti-platelet regimens and blood pressure control targets), leading to generalization bias of the model. Ethical and data acquisition barriers: Multicenter studies require the coordination of at least 8 hospitals’ ethics committees. Moreover, postoperative complication data are sensitive information, and the anonymization process may result in the loss of information on key variables (such as aneurysm location and embolization materials). In addition, some centers lack continuous blood pressure monitoring data, making it difficult to reproduce the key variable of ‘postoperative blood pressure fluctuations’. To address this critical limitation, we have initiated the China Multicenter Study on Intracranial Aneurysm Postoperative Complications (CHIPS study), which aims to enroll more than 500 patients from 8 hospitals across China. This study will conduct external validation of the current nomogram, adjust model parameters based on multi-center data, and improve its generalizability to different clinical settings. Ethical and data acquisition barriers: Multicenter studies require the coordination of at least 10 hospitals’ ethics committees. Moreover, postoperative complication data are sensitive information, and the anonymization process may result in the loss of information on key variables (such as aneurysm location and embolization materials) (19). In addition, some centers lack continuous blood pressure monitoring data, making it difficult to reproduce the key variable of “postoperative blood pressure fluctuations”. There are also other limitations in the study. Due to the significant inter-center heterogeneity in the clinical characteristics of patients with intracranial aneurysms (such as the proportion of posterior circulation aneurysms and the prevalence of hypertension), the efficacy of the model may decline when it is applied to other medical centers.

The core value of this study lies in providing a visual and quantifiable risk assessment tool. However, the following key points need to be noted for its clinical application: dynamic update of the model: it is recommended that clinicians calibrate the model parameters regularly by incorporating the latest research data (such as the impact of novel flow-diverting devices on the risk of rebleeding) when using the model (20). For example, with the popularization of flow-diverting devices, the definition of incomplete embolization may need to be re-defined, and the treatment modality should be incorporated into the model as an adjustment factor. Formulation of individualized intervention strategies: For high-risk patients, in addition to strict blood pressure control, early use of antifibrinolytic drugs (such as aminocaproic acid) can be considered to reduce the risk of rebleeding, or high-resolution vessel wall imaging (HR-VWI) can be used to evaluate the degree of aneurysm wall inflammation to guide anti-inflammatory treatment (17).

Another potential bias is that all patients were recruited from the neurosurgery department of a single center, which may limit the model’s generalizability to patients treated in other departments (e.g., interventional radiology). Additionally, this retrospective study has inherent selection bias: 28 patients with incomplete clinical records were excluded, which may have affected the representativeness of the study. To mitigate this, we performed a sensitivity analysis by excluding 15 additional patients with partially missing data (e.g., incomplete blood pressure records), and the model’s C-index remained stable (0.861 in the training set), indicating robustness to minor data missing. We confirm that all included patients had ruptured intracranial aneurysms, as evidenced by subarachnoid hemorrhage on pre-operative CT or DSA, so rupture status was not an adjusting variable in the regression model. Notably, antiplatelet use was significantly associated with early rebleeding in univariate analysis (*p* = 0.003) but excluded from the multivariate model due to confounding by embolization degree [variance inflation factor (VIF) = 3.92]. Patients with incomplete embolization were more likely to receive long-term antiplatelet therapy to reduce thrombotic complications, leading to collinearity that masked the independent effect of antiplatelet use itself. We have verified the publication status of references: Ling et al. (13) is an in-press article accepted by Clinical Neurology and Neurosurgery. We also added three recent studies (21–23) published in 2024, which confirmed the role of aneurysm wall inflammation (detected by HR-VWI) in rebleeding risk. Future iterations of the model will integrate HR-VWI-derived features (e.g., aneurysm wall enhancement grade, wall thickness) to capture biological characteristics of the aneurysm wall, further improving predictive accuracy.

Future research needs to make breakthroughs in three aspects: Conduct a multi-center study on postoperative complications of intracranial aneurysms in China (CHIPS study), including more than 500 patients, to validate the applicability of the model in different regions and ethnic groups. Another limitation is the relatively low event rate (22 rebleeding events in the training set) and potential overfitting risk. However, LASSO regression and bootstrap optimism correction have mitigated this risk, and power calculations confirmed the sample size was sufficient (power = 0.85, *α* = 0.05). Future multicenter studies (CHIPS study) with >500 patients will expand the event rate and improve generalizability. Integrate artificial intelligence image analysis technology to automatically extract morphological features of aneurysms (such as aneurysm body/neck ratio, degree of aneurysm wall calcification), and construct an “image-clinical” fusion model. Develop a real-time monitoring module to continuously monitor the tension of the aneurysm wall through implanted microsensors, and realize dynamic risk updates in combination with the nomogram, promoting the upgrade from “static prediction” to “dynamic warning.” The nomogram model constructed in this study systematically integrated the key risk factors for early rupture and rebleeding after intracranial aneurysm embolization for the first time. Through multivariate analysis, the synergistic mechanism of anatomical features, preoperative conditions, and postoperative management was revealed. Despite the limitation of a single-center study, the model showed good predictive efficacy in internal validation, providing the first quantitative assessment tool for clinical practice. In the future, multi-center data validation, biomarker integration, and digital transformation are required to further improve the universality and practicality of the model, ultimately achieving precise prevention and control of postoperative complications of intracranial aneurysms and opening up a new path for improving patient prognosis.

## Data Availability

The original contributions presented in the study are included in the article/supplementary material, further inquiries can be directed to the corresponding author.

## References

[1] Jin J Duan J Du L Xing W Peng X Zhao Q. Inflammation and immune cell abnormalities in intracranial aneurysm subarachnoid hemorrhage (SAH): relevant signaling pathways and therapeutic strategies. Front Immunol. (2022) 13:1027756. doi: 10.3389/fimmu.2022.1027756, 36505409 PMC9727248

[2] Aladawi M Elfil M Ghozy S Najdawi ZR Ghaith H Alzayadneh M . The impact of systolic blood pressure reduction on aneurysm re-bleeding in subarachnoid hemorrhage: a systematic review and meta-analysis. J Stroke Cerebrovasc Dis. (2024) 33:108084. doi: 10.1016/j.jstrokecerebrovasdis.2024.108084, 39395550

[3] Jia XF Chen YC Zheng KK Zhu DQ Chen C Liu J . Clinical-radiomics nomogram model based on CT angiography for prediction of intracranial aneurysm rupture: a multicenter study. J Multidiscip Healthc. (2024) 17:5917–26. doi: 10.2147/jmdh.S491697, 39678712 PMC11645942

[4] Ye Y Chen J Qiu X Chen J Ming X Wang Z . Prediction of small intracranial aneurysm rupture status based on combined clinical-radiomics model. Heliyon. (2024) 10:e30214. doi: 10.1016/j.heliyon.2024.e30214, 38707310 PMC11066671

[5] Wang Z Yan C Yuan W Jiang S Jiang Y Chen T. Enhancing intracranial aneurysm rupture risk prediction with a novel multivariable logistic regression model incorporating high-resolution vessel wall imaging. Front Neurol. (2024) 15:1507082. doi: 10.3389/fneur.2024.1507082, 39944838 PMC11816361

[6] An X He J Di Y Wang M Luo B Huang Y . Intracranial aneurysm rupture risk estimation with multidimensional feature fusion. Front Neurosci. (2022) 16:813056. doi: 10.3389/fnins.2022.813056, 35250455 PMC8893318

[7] Ouyang G Zheng KL Luo K Qiao M Zhu Y Pan D-R. Endovascular treatment of direct carotid cavernous fistula resulting from rupture of intracavernous carotid aneurysm: a case report. World J Clin Cases. (2024) 12:1940–6. doi: 10.12998/wjcc.v12.i11.1940, 38660547 PMC11036523

[8] Qin F Liu J Zhao X Wu D Lai N Zhang Z . Endovascular treatment of ruptured very small intracranial aneurysms: complications, recurrence rate, and clinical outcomes. Front Neurol. (2021) 12:767649. doi: 10.3389/fneur.2021.767649, 35058874 PMC8764134

[9] Uchikawa H Kin T Koizumi S Sato K Uchida T Takeda Y . Aneurysmal inflow rate coefficient predicts ultra-early rebleeding in ruptured intracranial aneurysms: preliminary report of a computational fluid dynamics study. Neurol Med Chir (Tokyo). (2023) 63:450–6. doi: 10.2176/jns-nmc.2023-0003, 37612121 PMC10687667

[10] Hostettler IC Lange N Schwendinger N Frangoulis S Hirle T Trost D . Duration between aneurysm rupture and treatment and its association with outcome in aneurysmal subarachnoid haemorrhage. Sci Rep. (2023) 13:1527. doi: 10.1038/s41598-022-27177-9, 36707604 PMC9883503

[11] Koiso T Komatsu Y Watanabe D Ikeda G Hosoo H Sato M . The influence of aneurysm size on the outcomes of endovascular management for aneurysmal subarachnoid hemorrhages: a comparison of the treatment results of patients with large and small aneurysms. Neurol Med Chir. (2023) 63:104–10. doi: 10.2176/jns-nmc.2022-0253, 36599431 PMC10072888

[12] Diestro JDB Dibas M Adeeb N Regenhardt RW Vranic JE Guenego A . Intrasaccular flow disruption for ruptured aneurysms: an international multicenter study. J Neurointerv Surg. (2023) 15:844–50. doi: 10.1136/jnis-2022-019153, 35868856

[13] Ling H Tao T Li W Zhuang Z Ding P Na S . Predictors of poor functional outcome after endovascular treatment in patients with poor-grade aneurysmal subarachnoid hemorrhage. Clin Neurol Neurosurg. (2025) 251:108792. doi: 10.1016/j.clineuro.2025.108792, 40054121

[14] Cheng R Su K Zhou X Jiang X Luo P Zhang W . Does dual antiplatelet therapy increase the risk of haematoma enlargement in the acute stage? A retrospective study of the use of stent-assisted coiling versus coiling alone or balloon-assisted coiling for the treatment of ruptured intracranial aneurysms combined with intracranial haematoma. Neurosurg Rev. (2023) 46:133. doi: 10.1007/s10143-023-02036-x, 37266675

[15] Ren D Li J Zhou B Guo S Guo B. Modeling of the dynamics of vascular embolization by using porous media for the design of injection robots of embolic agents. Med Eng Phys. (2022) 101:103774. doi: 10.1016/j.medengphy.2022.103774, 35232547

[16] Brunozzi D See A Rizko M Choi J Atwal G Alaraj A. Impact of cerebral aneurysm size on distal intracranial hemodynamics and changes following flow diversion. Interv Neuroradiol. (2022) 28:291–5. doi: 10.1177/15910199211032467, 34425691 PMC9185094

[17] Dugani SM. Management of intraoperative rupture of intracranial aneurysms: agony and ecstasy. Acta Neurochir Suppl. (2023) 130:65–79. doi: 10.1007/978-3-030-12887-6_9, 37548725

[18] de Winkel J Roozenbeek B Dijkland SA Dammers R van Doormaal PJ van der Jagt M . Personalized decision-making for aneurysm treatment of aneurysmal subarachnoid hemorrhage: development and validation of a clinical prediction tool. BMC Neurol. (2024) 24:65. doi: 10.1186/s12883-024-03546-x, 38360580 PMC10868110

[19] Ten Brinck MFM Shimanskaya VE Aquarius R Bartels RHMA Meijer FJA Koopmans PC . Outcomes after flow diverter treatment in subarachnoid hemorrhage: a meta-analysis and development of a clinical prediction model (outflow). Brain Sci. (2022) 12:394. doi: 10.3390/brainsci12030394, 35326350 PMC8946659

[20] Lepine HL Semione G Llata FM Nogueira BV Pereira ACPG Coelho DN . Treatment of ruptured intracranial aneurysms with parent artery flow diverter devices: a comprehensive systematic review and meta-analysis. Int J Stroke. (2024) 20:17474930241307114. doi: 10.1177/17474930241307114, 39614729

[21] Zwarzany Ł Sawicki M Poncyljusz W. Significance of aneurysm wall enhancement on high-resolution vessel wall magnetic resonance imaging in clinical management of patients with intracranial aneurysms. Neurol Neurochir Pol. (2020) 54:518–23. doi: 10.5603/Pjnns.a2020.0087, 33089880

[22] Fu Q Zhang Y Zhang Y Liu C Li J Wang M . Wall permeability on magnetic resonance imaging is associated with intracranial aneurysm symptoms and wall enhancement. Eur Radiol. (2024) 34:5204–14. doi: 10.1007/s00330-023-10548-9, 38224377 PMC11247137

[23] Omodaka S Si S Sakata H Funamoto K Yamaguchi T Niizuma K . Aneurysm Wall enhancement can predict rupture point in intracranial aneurysms with multiple blebs. Neurosurgery. (2025) 96:593–9. doi: 10.1227/neu.0000000000003134, 39115321

